# Mechanisms guiding Polycomb activities during gene silencing in *Arabidopsis thaliana*

**DOI:** 10.3389/fpls.2013.00454

**Published:** 2013-11-13

**Authors:** Chongsheng He, Hai Huang, Lin Xu

**Affiliations:** National Laboratory of Plant Molecular Genetics, Shanghai Institute of Plant Physiology and Ecology, Shanghai Institutes for Biological Sciences, Chinese Academy of SciencesShanghai, China

**Keywords:** *Arabidopsis*, epigenetics, histone modification, Polycomb group, Polycomb Repressive Complex, Trithorax group

## Abstract

Polycomb group (PcG) proteins act in an evolutionarily conserved epigenetic pathway that regulates chromatin structures in plants and animals, repressing many developmentally important genes by modifying histones. PcG proteins can form at least two multiprotein complexes: Polycomb Repressive Complexes 1 and 2 (PRC1 and PRC2, respectively). The functions of *Arabidopsis thaliana* PRCs have been characterized in multiple stages of development and have diverse roles in response to environmental stimuli. Recently, the mechanism that precisely regulates *Arabidopsis* PcG activity was extensively studied. In this review, we summarize recent discoveries in the regulations of PcG at the three different layers: the recruitment of PRCs to specific target loci, the polyubiquitination and degradation of PRC2, and the antagonism of PRC2 activity by the Trithorax group proteins. Current knowledge indicates that the powerful activity of the PcG pathway is strictly controlled for specific silencing of target genes during plant development and in response to environmental stimuli.

## INTRODUCTION TO POLYCOMB REPRESSIVE COMPLEXES IN *ARABIDOPSIS THALIANA*

Multicellular eukaryotic organisms develop from a single cell called a zygote, which goes through cell division and differentiation to develop into multiple tissues and organs. Precise control of gene expression under the guidance of developmental cues/signals or environmental stimuli is strictly regulated to ensure proper development of organisms. Plants and animals have evolved to have multiple methods to regulate gene expression, among which epigenetic regulation is essential for correct genome-wide gene expression profiles (Russo et al., 1996; Liu et al., 2010a).

Polycomb group (PcG) proteins, one of the evolutionarily conserved epigenetic pathways, have critical roles in plant and animal development via regulation of gene expression levels (Whitcomb et al., 2007; Molitor and Shen, 2013). In the model plant* Arabidopsis thaliana*, PcG proteins are incorporated into two multi-protein complexes: Polycomb Repressive Complexes 1 and 2 (PRC1 and PRC2, respectively), which both have functions in the epigenetic repression of gene expression via histone modifications (Schatlowski et al., 2008; Molitor and Shen, 2013). The major function of PRC2 is to trimethylate lysine 27 on histone H3 (H3K27me3), while PRC1 recognizes the H3K27me3 marker and mono-ubiquitinates histone H2A (H2Aub; Schatlowski et al., 2008; Molitor and Shen, 2013).

The core component of *Arabidopsis *PRC2 is the SET-domain H3K27 methyltransferase CURLY LEAF (CLF; Goodrich et al., 1997; Schubert et al., 2006). In addition, SWINGER (SWN) has partially redundant functions with CLF (Chanvivattana et al., 2004), and MEDEA (MEA) functions as a methyltransferase in the endosperm (Grossniklaus et al., 1998). Some other assistant proteins are also required to form PRC2, such as EMBRYONIC FLOWER2 (EMF2; Yoshida et al., 2001). There are about 4,400 gene loci that are modified by H3K27me3 in *Arabidopsis* seedlings (Zhang et al., 2007a). Such modifications are dynamic and the modification patterns differ among organs and tissues. Genome-wide identification of H3K27me3-modificed loci revealed that this dynamic modification occurs during the shoot apical meristem (SAM)-to-leaf organ formation (Lafos et al., 2011), the embryo-to-seedling phase transition (Bouyer et al., 2011), in leaf-to-callus regeneration (He et al., 2012), and in the endosperm (Weinhofer et al., 2010). Therefore, PRC2-mediated H3K27me3 is precisely controlled during development.

PRC1 components have also been characterized in *Arabidopsis*. LIKE HETEROCHROMATIN PROTEIN1 (LHP1), also called TERMINAL FLOWER2 (TFL2), can recognize chromatin that is modified by H3K27me3, and its genome-wide binding sites show significant overlap with the H3K27me3 modification (Turck et al., 2007; Zhang et al., 2007b). There are two groups of RING-domain proteins in PRC1: two AtRING1 proteins and three AtBMI1 proteins (Xu and Shen, 2008; Bratzel et al., 2010; Chen et al., 2010). These RING-domain proteins function in the modification of H2Aub (Bratzel et al., 2010). Although the genome-wide identification of H2Aub loci has not been performed, it is possible that the role of PRC1 is also developmentally controlled (Kim et al., 2012).

## RECRUITMENT OF PRCS TO SPECIFIC TARGET GENES

How PRCs specifically and dynamically recognize their targets during development and in different tissues or organs is a key question for understanding the mechanism that controls the PcG pathway. Currently, there are two types of recruiters found in *Arabidopsis*: transcription factors and non-coding RNAs (ncRNAs).

Transcription factors usually bind to certain specific *cis* elements, termed Polycomb response elements (PREs), and recruit PRCs to their specific targets via direct interaction with PRCs (Schwartz and Pirrotta, 2008). AGAMOUS (AG), a MADS-box transcription factor that is essential for establishing floral organ identity and termination of floral meristem (FM), represses *WUSCHEL* (*WUS*) expression in FM (Bowman et al., 1989; Lenhard et al., 2001; Lohmann et al., 2001). AG binds to the *WUS* locus at CArG boxes and then recruits PRC2-mediated H3K27me3 and LHP1 to repress *WUS* (Liu et al., 2011). Mutation in *AG* results in the decreased H3K27me3 level and loss of LHP1 binding at the *WUS* locus. In this case, CArG boxes may serve as the PRE in *WUS* repression. However, it is not clear whether AG directly or indirectly recruits PcG proteins.

*BREVIPEDICELLUS *(*BP*) and *KNOTTED-LIKE FROM ARABIDOPSIS THALIANA2 *(*KNAT2*) are members of the KNOX gene family and are expressed in the SAM but are silenced in leaves (Lincoln et al., 1994; Ori et al., 2000; Pautot et al., 2001). The MYB-domain transcription factor ASYMMETRIC LEAVES1 (AS1) and the LOB-domain transcription factor AS2 form a protein complex to repress *BP* and *KNAT2* expression in leaves (Byrne et al., 2000; Ori et al., 2000; Semiarti et al., 2001; Iwakawa et al., 2002; Sun et al., 2002; Lin et al., 2003; Xu et al., 2003). The AS1-AS2 complex targets the *cis* elements in the promoters of *BP* and* KNAT2* (Guo et al., 2008; Li et al., 2012). Recently, it was shown that AS1-AS2 interacts with PRC2 to recruit it to the *BP* and *KNAT2* loci (Lodha et al., 2013). Mutations in *AS1*, *AS2*, and the AS1-AS2-binding sites in *BP* and* KNAT2* promoters all result in decreased H3K27me3 levels at *BP* and *KNAT2* loci and ectopic expression of the two KNOX genes in leaves. In this case, the AS1-AS2-binding sites are likely to serve as PREs in PcG-mediated gene silencing.

Another possible PRE was found in the promoter of *LEAFY COTYLEDON2 *(*LEC2*; Berger et al., 2011), a gene involved in embryo development, but which is silenced in the post-embryo stage (Stone et al., 2001). A *cis* element, called *Repressive LEC2 Element *(*RLE*), was identified to recruit PRC2 to trimethylate the *LEC2* locus (Berger et al., 2011). *RLE* is essential for *LEC2* repression in the post-embryo stage. PRC2 is unable to trimethylate H3K27 at the *LEC2* locus once *RLE* is mutated, leading to ectopic expression of *LEC2* in the post-embryo stage. An *RLE-*driven reporter gene could be repressed, accompanied by H3K27me3 modification at the transgene locus, suggesting that *RLE *is necessary and sufficient to recruit PRC2 for histone modification and gene silencing. It seems important in the future to identify the transcription factor that binds *RLE* and is able to interact with PRC2.

An analysis of *cis*-regulatory elements in the promoter of *FLOWERING LOCUS T* (*FT*), a key gene that controls flowering time (Kardailsky et al., 1999; Kobayashi et al., 1999), also indicated the existence of PRE within the promoter region (Adrian et al., 2010). However, the exact sequence that serves as the PRE and the transcription factor that binds the PRE of the *FT* promoter have not yet been identified.

The B3 domain proteins VP1/ABI3-LIKE1 (VAL1) and VAL2 are key regulators in the prevention of embryo traits in somatic tissues via repression of embryo specific genes. The loss-of-function double mutant *val1 val2* results in somatic embryogenesis, with ectopic expressions of embryo genes at the post-embryo stage (Suzuki et al., 2007). Somatic embryogenesis and ectopic expression of embryo genes were also observed in mutants corresponding to PRC1 components (Bratzel et al., 2010; Chen et al., 2010). A recent study showed that VAL proteins interact with PRC1 and recruit PRC1-mediated H2Aub to initiate repression of the embryonic genes after germination (Yang et al., 2013). The H2Aub modification at the embryo-gene loci is lost in* val1 val2* and *Atbmi1a Atbmi1b *mutants. Therefore, VALs may serve as a recruiter for PRC1.

In *Arabidopsis*, two ncRNAs, *COLD INDUCED LONG ANTISENSE INTRAGENIC RNA *(*COOLAIR*)**and *COLD ASSISTED INTRONIC NONCODING RNA* (*COLDAIR*; Swiezewski et al., 2009; Heo and Sung, 2011; Ietswaart et al., 2012),**regulate *FLOWERING LOCUS C* (*FLC*) expression. *FLC *is a flowering repressor that is essential for vernalization in response to cold treatment (Michaels and Amasino, 1999; Sheldon et al., 1999). *COOLAIR* is an antisense RNA that is transcribed in response to cold treatment (Swiezewski et al., 2009). *COOLAIR* is alternatively polyadenylated at the 3′ end, resulting in a proximal poly(A) site or a distal ploy(A) site (Liu et al., 2010b). The proximal poly(A) site stimulates the activity of FLD, a homolog of the human LYSINE SPECIFIC DEMETHYLASE1 (LSD1; Sanda and Amasino, 1996; Liu et al., 2007), to reduce the H3K4me2 level at the *FLC* locus, leading to a transition from an active chromatin state to a repressive state (Liu et al., 2010b). The reduction of H3K4me2 might benefit the H3K27me3 modification; thus, *COOLAIR* acts as an indirect recruiter of PRC2. However, how FLD is activated using the proximal site of *COOLAIR* remains unknown.

*COLDAIR* is a sense ncRNA that contains a 5′ cap, but no poly(A) tail (Heo and Sung, 2011). *COLDAIR* is induced by low temperature, and its transcription reaches a maximum level after 20 days of cold treatment, which is about 10 days later than *COOLAIR*. *COLDAIR* can directly interact with the CXC domain of the core PRC2 components CLF and SWN. In *COLDAIR* knockdown plants, CLF is not properly recruited to *FLC*, resulting in insufficient H3K27me3 modification at the *FLC* locus, consistent with the late flowering phenotype of these plants. Therefore, COLDAIR serves as a direct recruiter for PRC2 in *Arabidopsis*.

### PRC2 DEGRADATION THROUGH POLYUBIQUITINATION

The *Arabidopsis* PRC2 is post-translationally regulated by the F-box protein UPWARD CURLY LEAF1 (UCL1; Jeong et al., 2011). UCL1 is a component of the SCF E3 ubiquitin ligase complex, which has a role in the degradation of proteins via polyubiquitination and the 26S proteasome pathway (Vierstra, 2003). Both activation tagging dominant mutant *ucl1-D*, and plants overexpressing *UCL1* under the control of the CaMV 35S promoter resulted in phenotypes that were similar to the *clf* mutant, with ectopic expression of some typical PRC2 targets, whose loci exhibit decreased H3K27me3 levels (Jeong et al., 2011). These results indicated that UCL1 inhibits PRC2 activity. Additionally, UCL1 directly interacts with CLF, but not with MEA, and overexpression of *UCL1* causes a reduced CLF protein level. Therefore, UCL1 was thought to polyubiquitinate CLF and to degrade CLF through the 26S proteasome pathway.

Interestingly, *UCL1* is expressed in the endosperm, where *CLF* and *MEA* transcripts are detectable. However, overexpression of *CLF *in the endosperm causes *mea*-like phenotypes, suggesting that the CLF protein level is strictly controlled in the endosperm. Jeong et al. suggested that UCL1 functions in the endosperm to specifically degrade CLF, and therefore to prevent CLF from competing with MEA during the formation of PRC2 (Jeong et al., 2011).

## PRC2 FUNCTIONS ARE ANTAGONIZED BY THE TRITHORAX GROUP PROTEINS

Trithorax group (TrxG) proteins were first identified in *Drosophila* and function in antagonism of PcG (Schuettengruber et al., 2011). The first identified plant TrxG protein was *Arabidopsis* HOMOLOG OF TRITHORAX1 (ATX1), which encodes a SET-domain protein that specifically trimethylates H3K4 (Alvarez-Venegas et al., 2003; Pien et al., 2008; Saleh et al., 2008). Two other SET-domain proteins were shown to participate in genome-wide control of histone methylation: SDG8 is responsible for H3K36me2/3 (Zhao et al., 2005; Xu et al., 2008), and SDG2 acts for H3K4me2/3 (Berr et al., 2010; Guo et al., 2010). The histone modifications H3K4me2/3 and H3K36me2/3 function in activation of gene expression, showing the opposite role of PcG-mediated H3K27me3 (Zhang et al., 2007a, 2009; Roudier et al., 2011). However, the molecular mechanism whereby H3K4me2/3 and H3K36me2/3 antagonize H3K27me3 is not clear in *Arabidopsis*. A recent study showed that the 35S enhancer decreases the H3K27me3 level but increases the H3K4me3 level at the insert locus (Chen et al., 2013). This suggests that *cis* enhancer sequences may play a role to recruit TrxG proteins to restrict the PRC2-mediated H3K27me3.

ULTRAPETALA1 (ULT1), a plant-specific SAND domain protein, is another TrxG protein (Carles and Fletcher, 2009). Overexpression of *ULT1* resulted in phenotypes similar to those of *clf*, and caused derepression of PcG target genes with decreased H3K27me3 levels. ULT1 is able to directly bind to ATX1 and guides it to the target genes.

RELATIVE OF EARLY FLOWERING6 (REF6) is a JmjC-domain protein (Noh et al., 2004) that specifically demethylates H3K27me2/3 *in vivo* and *in vitro *(Lu et al., 2011). Overexpression of *REF6* caused an* lhp1*-like phenotype (Lu et al., 2011). In addition, mutation of *REF6* could partially rescue the *clf* phenotype, and resulted in hypermethylation of H3K27me3 for hundreds of genes. Characterizationof REF6 revealed that the H3K27me3 modification is a reversible process, and such regulation is critical to balance PcG activity (Lu et al., 2011).

The ATP-dependent chromatin remodeling factor PICKLE (PKL) is involved in antagonism of PcG (Aichinger et al., 2009, 2011), and the *pkl* mutant partially rescued developmental defects of the *clf* mutant, including the up-curled leaves and early flowering. Conversely, *pkl* enhanced the defects of the *clf swn* double mutant in the root. In roots, PKL activates the expression of *EMF2*, * CLF*, and*SWN*, and this could explain why *pkl *enhances* clf swn. *In addition,**PKL is very important for the activity of the root apical meristem (RAM). In the *pkl* mutant, a number of genes that respond to the activity of RAM were silenced because of the increased H3K27me3 level on these genes (Aichinger et al., 2011). However, several other studies suggested that PKL has a role in the promotion of PRC2-mediated H3K27me3 and that the PKL protein associates with H3K27me3-enriched loci (Zhang et al., 2008, 2012). Further studies to clarify the genome-wide function of PKL will improve our understanding of how the chromatin remodeling pathway coordinates with the PcG pathway to regulate their downstream targets. Overall, these studies revealed that chromatin structure, which is organized by chromatin remodeling factors using energy from ATP hydrolysis, is essential for regulation of histone modification states.

## CONCLUSION AND PERSPECTIVES

**FIGURE 1:**
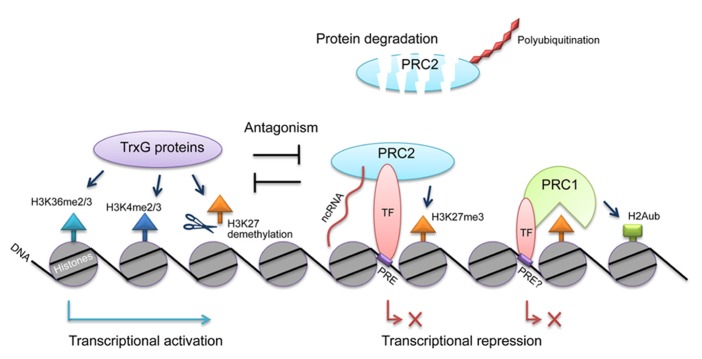
**Regulation of PcG actions by transcription factors, ncRNAs, polyubiquitination, and TrxG proteins.** PcG complexes PRC1 and PRC2 can be recruited to specific loci by transcription factors (TF) and ncRNAs. The PRC2 component CLF is post-translationally regulated by polyubiquitination, leading to protein degradation. PcG and TrxG antagonize each other to reach a dynamic regulation of gene expression.

Controls for the PcG action at different levels are essential to ensure its specificity and activity (see the model in **Figure 1**). However, many questions still remain to be answered. First, although several candidates that recruit PRC1 and PRC2 to the target genes have been characterized, the common rule of the recruitment remains unclear. For example, how do these transcription factors bind PRCs? If PRCs are recruited generally by transcription factors, common domains or motifs might exist in the PcG proteins and transcription factors, similar to the binding of the TOPLESS corepressor and transcription factors (Causier et al., 2012). On the other hand, if ncRNAs are the common factors that associate with PRCs, the RNA-binding domain of PcG proteins may contain various ncRNAs. Detection of these ncRNAs might be helpful in studying the specificity of PcG. In addition, how the ncRNAs are transcribed in response to developmental or environmental cues is not clear. Recently, it was proposed that the chromatin structure and its modification status also affect the recruitment of PRCs to target loci in the “chromatin sampling” model (Klose et al., 2013), and it was also shown that PRC2 binding sites contain GAGA factor binding sequences (Deng et al., 2013).

Second, the mechanism guiding the balance between PcG and TrxG activities in regulating developmental processes in plants is currently unknown. In animals, several transcription factors can regulate both PcG and TrxG activity, providing a dynamic and reversible epigenetic state (Schwartz and Pirrotta, 2008). Therefore, comparison of the regulation mechanisms of PcG and TrxG may be helpful to understand how a balance is established between PcG-mediated gene silencing and TrxG-mediated gene activation.

Third, some novel proteins were identified in plant PRCs, for example, the CLF interacting protein BLISTER (BLI; Schatlowski et al., 2010) and LHP1-INTERACTING FACTOR2 (LIF2; Latrasse et al., 2011). It will be of interest to test whether these proteins act as regulators of PRCs. LIF2 is an RNA-binding protein, suggesting that PRC1 may also be subject to regulation from RNAs.

Finally, recent studies revealed that PRC1 and PRC2 physically interact in *Arabidopsis* (Derkacheva et al., 2013), indicating the possibility that the two PcG complexes have a crosstalk in silencing common targets. Further studies on mechanisms that regulate PcG activity would be helpful to understand the epigenetic regulation of plant development.

## Conflict of Interest Statement

The authors declare that the research was conducted in the absence of any commercial or financial relationships that could be construed as a potential conflict of interest.

## References

[B1] Adrian J. Farrona S. Reimer J. J. Albani M. C. Coupland G. Turck F. (2010). cis-Regulatory elements and chromatin state coordinately control temporal and spatial expression of FLOWERING LOCUS T in *Arabidopsis*. *Plant Cell* 22 1425–1440 10.1105/tpc.110.074682 20472817 PMC2899882

[B2] Aichinger E. Villar C. B. Di Mambro R. Sabatini S. Kohler C. (2011). The CHD3 chromatin remodeler PICKLE and polycomb group proteins antagonistically regulate meristem activity in the *Arabidopsis* root. *Plant Cell* 23 1047–1060 10.1105/tpc.111.083352 21441433 PMC3082253

[B3] Aichinger E. Villar C. B. Farrona S. Reyes J. C. Hennig L. Kohler C. (2009). CHD3 proteins and polycomb group proteins antagonistically determine cell identity in *Arabidopsis*. *PLoS Genet.* 5:e1000605 10.1371/journal.pgen.1000605 PMC2718830 19680533

[B4] Alvarez-Venegas R. Pien S. Sadder M. Witmer X. Grossniklaus U. Avramova Z. (2003). ATX-1, an *Arabidopsis* homolog of trithorax, activates flower homeotic genes. *Curr. Biol.* 13 627–637 10.1016/S0960-9822(03)00243-4 12699618

[B5] Berger N. Dubreucq B. Roudier F. Dubos C. Lepiniec L. (2011). Transcriptional regulation of *Arabidopsis* LEAFY COTYLEDON2 involves RLE, a cis-element that regulates trimethylation of histone H3 at lysine-27. *Plant Cell* 23 4065–4078 10.1105/tpc.111.087866 22080598 PMC3246333

[B6] Berr A. Mccallum E. J. Menard R. Meyer D. Fuchs J. Dong A. (2010). *Arabidopsis* SET DOMAIN GROUP2 is required for H3K4 trimethylation and is crucial for both sporophyte and gametophyte development. *Plant Cell* 22 3232–3248 10.1105/tpc.110.079962 21037105 PMC2990135

[B7] Bouyer D. Roudier F. Heese M. Andersen E. D. Gey D. Nowack M. K. (2011). Polycomb repressive complex 2 controls the embryo-to-seedling phase transition. *PLoS Genet.* 7:e1002014 10.1371/journal.pgen.1002014 PMC3053347 21423668

[B8] Bowman J. L. Smyth D. R. Meyerowitz E. M. (1989). Genes directing flower development in *Arabidopsis*. *Plant Cell* 1 37–52 10.1105/tpc.1.1.37 2535466 PMC159735

[B9] Bratzel F. Lopez-Torrejon G. Koch M. Del Pozo J. C. Calonje M. (2010). Keeping cell identity in *Arabidopsis* requires PRC1 RING-finger homologs that catalyze H2A monoubiquitination. *Curr. Biol.* 20 1853–1859 10.1016/j.cub.2010.09.046 20933424

[B10] Byrne M. E. Barley R. Curtis M. Arroyo J. M. Dunham M. Hudson A. (2000). Asymmetric leaves1 mediates leaf patterning and stem cell function in *Arabidopsis*. *Nature* 408 967–971 10.1038/35050091 11140682

[B11] Carles C. C. Fletcher J. C. (2009). The SAND domain protein ULTRAPETALA1 acts as a trithorax group factor to regulate cell fate in plants. *Genes Dev.* 23 2723–2728 10.1101/gad.1812609 19952107 PMC2788324

[B12] Causier B. Ashworth M. Guo W. Davies B. (2012). The TOPLESS interactome: a framework for gene repression in *Arabidopsis*. *Plant Physiol.* 158 423–438 10.1104/pp.111.186999 22065421 PMC3252085

[B13] Chanvivattana Y. Bishopp A. Schubert D. Stock C. Moon Y. H. Sung Z. R. (2004). Interaction of Polycomb-group proteins controlling flowering in *Arabidopsis*. *Development* 131 5263–5276 10.1242/dev.01400 15456723

[B14] Chen X. Huang H. Xu L. (2013). The CaMV 35S enhancer has a function to change the histone modification state at insertion loci in *Arabidopsis thaliana*. *J. Plant Res*. In press 10.1007/s10265-013-0580-4 23880941

[B15] Chen D. Molitor A. Liu C. Shen W. H. (2010). The *Arabidopsis* PRC1-like ring-finger proteins are necessary for repression of embryonic traits during vegetative growth. *Cell Res.* 20 1332–1344 10.1038/cr.2010.151 21060339

[B16] Deng W. Buzas D. M. Ying H. Robertson M. Taylor J. Peacock W. J. (2013). *Arabidopsis* Polycomb Repressive Complex 2 binding sites contain putative GAGA factor binding motifs within coding regions of genes. *BMC Genomics* 14:593 10.1186/1471-2164-14-593 PMC3766684 24001316

[B17] Derkacheva M. Steinbach Y. Wildhaber T. Mozgova I. Mahrez W. Nanni P. (2013). *Arabidopsis* MSI1 connects LHP1 to PRC2 complexes. *EMBO J.* 32 2073–2085 10.1038/emboj.2013.145 23778966 PMC3715863

[B18] Goodrich J. Puangsomlee P. Martin M. Long D. Meyerowitz E. M. Coupland G. (1997). A Polycomb-group gene regulates homeotic gene expression in *Arabidopsis*. *Nature* 386 44–51 10.1038/386044a0 9052779

[B19] Grossniklaus U. Vielle-Calzada J. P. Hoeppner M. A. Gagliano W. B. (1998). Maternal control of embryogenesis by MEDEA, a polycomb group gene in *Arabidopsis*. *Science* 280 446–450 10.1126/science.280.5362.446 9545225

[B20] Guo M. Thomas J. Collins G. Timmermans M. C. (2008). Direct repression of KNOX loci by the ASYMMETRIC LEAVES1 complex of *Arabidopsis*. *Plant Cell* 20 48–58 10.1105/tpc.107.056127 18203921 PMC2254922

[B21] Guo L. Yu Y. Law J. A. Zhang X. (2010). SET DOMAIN GROUP2 is the major histone H3 lysine 4 trimethyltransferase in *Arabidopsis*. *Proc. Natl. Acad. Sci. U.S.A*. 107 18557–18562 10.1073/pnas.1010478107 20937886 PMC2972934

[B22] He C. Chen X. Huang H. Xu L. (2012). Reprogramming of H3K27me3 is critical for acquisition of pluripotency from cultured *Arabidopsis* tissues. *PLoS Genet.* 8:e1002911 10.1371/journal.pgen.1002911 PMC3426549 22927830

[B23] Heo J. B. Sung S. (2011). Vernalization-mediated epigenetic silencing by a long intronic noncoding RNA. *Science* 331 76–79 10.1126/science.1197349 21127216

[B24] Ietswaart R. Wu Z. Dean C. (2012). Flowering time control: another window to the connection between antisense RNA and chromatin. *Trends Genet.* 28 445–453 10.1016/j.tig.2012.06.002 22785023

[B25] Iwakawa H. Ueno Y. Semiarti E. Onouchi H. Kojima S. Tsukaya H. (2002). The ASYMMETRIC LEAVES2 gene of *Arabidopsis* *thaliana,* required for formation of a symmetric flat leaf lamina, encodes a member of a novel family of proteins characterized by cysteine repeats and a leucine zipper. *Plant Cell Physiol.* 43 467–478 10.1093/pcp/pcf077 12040093

[B26] Jeong C. W. Roh H. Dang T. V. Choi Y. D. Fischer R. L. Lee J. S. (2011). An E3 ligase complex regulates SET-domain polycomb group protein activity in *Arabidopsis* *thaliana*. *Proc. Natl. Acad. Sci. U.S.A.* 108 8036–8041 10.1073/pnas.1104232108 21518870 PMC3093496

[B27] Kardailsky I. Shukla V. K. Ahn J. H. Dagenais N. Christensen S. K. Nguyen J. T. (1999). Activation tagging of the floral inducer FT. *Science* 286 1962–1965 10.1126/science.286.5446.1962 10583961

[B28] Kim S. Y. Lee J. Eshed-Williams L. Zilberman D. Sung Z. R. (2012). EMF1 and PRC2 cooperate to repress key regulators of Arabidopsis development. *PLoS Genet.* 8:e1002512 10.1371/journal.pgen.1002512 PMC3310727 22457632

[B29] Klose R. J. Cooper S. Farcas A. M. Blackledge N. P. Brockdorff N. (2013). Chromatin sampling-an emerging perspective on targeting polycomb repressor proteins. *PLoS Genet.* 9:e1003717 10.1371/journal.pgen.1003717 PMC3749931 23990804

[B30] Kobayashi Y. Kaya H. Goto K. Iwabuchi M. Araki T. (1999). A pair of related genes with antagonistic roles in mediating flowering signals. *Science* 286 1960–1962 10.1126/science.286.5446.1960 10583960

[B31] Lafos M. Kroll P. Hohenstatt M. L. Thorpe F. L. Clarenz O. Schubert D. (2011). Dynamic regulation of H3K27 trimethylation during *Arabidopsis* differentiation. *PLoS Genet.* 7:e1002040 10.1371/journal.pgen.1002040 PMC3072373 21490956

[B32] Latrasse D. Germann S. Houba-Herin N. Dubois E. Bui-Prodhomme D. Hourcade D. (2011). Control of flowering and cell fate by LIF2, an RNA binding partner of the polycomb complex component LHP1. *PLoS ONE* 6:e16592 10.1371/journal.pone.0016592 PMC3031606 21304947

[B33] Lenhard M. Bohnert A. Jurgens G. Laux T. (2001). Termination of stem cell maintenance in *Arabidopsis* floral meristems by interactions between *Wuschel* and *Agamous*. *Cell* 105 805–814 10.1016/S0092-8674(01)00390-7 11440722

[B34] Li Z. Li B. Shen W.-H. Huang H. Dong A. (2012). TCP transcription factors interact with AS2 in the repression of class-I KNOX genes in *Arabidopsis thaliana.* *Plant J.* 71 99–107 10.1111/j.1365-313X.2012.04973.x 22380849

[B35] Lin W. C. Shuai B. Springer P. S. (2003). The *Arabidopsis* LATERAL ORGAN BOUNDARIES-domain gene ASYMMETRIC LEAVES2 functions in the repression of KNOX gene expression and in adaxial-abaxial patterning. *Plant Cell* 15 2241–2252 10.1105/tpc.014969 14508003 PMC197291

[B36] Lincoln C. Long J. Yamaguchi J. Serikawa K. Hake S. (1994). A knotted1-like homeobox gene in *Arabidopsis* is expressed in the vegetative meristem and dramatically alters leaf morphology when overexpressed in transgenic plants. *Plant Cell* 6 1859–1876 10.1105/tpc.6.12.1859 7866029 PMC160567

[B37] Liu X. Kim Y. J. Muller R. Yumul R. E. Liu C. Pan Y. (2011). AGAMOUS terminates floral stem cell maintenance in *Arabidopsis* by directly repressing *Wuschel* through recruitment of polycomb group proteins. *Plant Cell* 23 3654–3670 10.1105/tpc.111.091538 22028461 PMC3229141

[B38] Liu C. Lu F. Cui X. Cao X. (2010a). Histone methylation in higher plants. *Annu. Rev. Plant Biol.* 61 395–420 10.1146/annurev.arplant.043008.091939 20192747

[B39] Liu F. Marquardt S. Lister C. Swiezewski S. Dean C. (2010b). Targeted 3^′^ processing of antisense transcripts triggers *Arabidopsis* FLC chromatin silencing. *Science* 327 94–97 10.1126/science.1180278 19965720

[B40] Liu F. Quesada V. Crevillen P. Baurle I. Swiezewski S. Dean C. (2007). The *Arabidopsis* RNA-binding protein FCA requires a lysine-specific demethylase 1 homolog to downregulate FLC. *Mol. Cell* 28 398–407 10.1016/j.molcel.2007.10.018 17996704

[B41] Lodha M. Marco C. F. Timmermans M. C. (2013). The ASYMMETRIC LEAVES complex maintains repression of KNOX homeobox genes via direct recruitment of Polycomb-repressive complex2. *Genes Dev.* 27 596–601 10.1101/gad.211425.112 23468429 PMC3613607

[B42] Lohmann J. U. Hong R. L. Hobe M. Busch M. A. Parcy F. Simon R. (2001). A molecular link between stem cell regulation and floral patterning in *Arabidopsis*. *Cell* 105 793–803 10.1016/S0092-8674(01)00384-1 11440721

[B43] Lu F. Cui X. Zhang S. Jenuwein T. Cao X. (2011). *Arabidopsis* REF6 is a histone H3 lysine 27 demethylase. *Nat. Genet.* 43 715–719 10.1038/ng.854 21642989

[B44] Michaels S. D. Amasino R. M. (1999). FLOWERING LOCUS C encodes a novel MADS domain protein that acts as a repressor of flowering. *Plant Cell* 11 949–956 10.1105/tpc.11.5.949 10330478 PMC144226

[B45] Molitor A. Shen W. H. (2013). The polycomb complex PRC1: composition and function in plants. *J. Genet. Genomics* 40 231–238 10.1016/j.jgg.2012.12.005 23706298

[B46] Noh B. Lee S. H. Kim H. J. Yi G. Shin E. A. Lee M. (2004). Divergent roles of a pair of homologous jumonji/zinc-finger-class transcription factor proteins in the regulation of *Arabidopsis* flowering time. *Plant Cell* 16 2601–2613 10.1105/tpc.104.025353 15377760 PMC520958

[B47] Ori N. Eshed Y. Chuck G. Bowman J. L. Hake S. (2000). Mechanisms that control knox gene expression in the *Arabidopsis* shoot. *Development* 127 5523–5532 11076771 10.1242/dev.127.24.5523

[B48] Pautot V. Dockx J. Hamant O. Kronenberger J. Grandjean O. Jublot D. (2001). KNAT2: evidence for a link between knotted-like genes and carpel development. *Plant Cell* 13 1719–1734 11487688 10.1105/TPC.010184 PMC139140

[B49] Pien S. Fleury D. Mylne J. S. Crevillen P. Inze D. Avramova Z. (2008). *ARABIDOPSIS TRITHORAX1* dynamically regulates FLOWERING LOCUS C activation via histone 3 lysine 4 trimethylation. *Plant Cell* 20 580–588 10.1105/tpc.108.058172 18375656 PMC2329943

[B50] Roudier F. Ahmed I. Berard C. Sarazin A. Mary-Huard T. Cortijo S. (2011). Integrative epigenomic mapping defines four main chromatin states in *Arabidopsis*. *EMBO J.* 30 1928–1938 10.1038/emboj.2011.103 21487388 PMC3098477

[B51] Russo V. E. A. Martienssen R. A. Riggs A. D. (1996). *Epigenetic Mechanisms of Gene Regulation*. Woodbury: Cold Spring Harbor Laboratory Press

[B52] Saleh A. Alvarez-Venegas R. Yilmaz M. Le O. Hou G. Sadder M. (2008). The highly similar *Arabidopsis* homologs of trithorax ATX1 and ATX2 encode proteins with divergent biochemical functions. *Plant Cell* 20 568–579 10.1105/tpc.107.056614 18375658 PMC2329920

[B53] Sanda S. L. Amasino R. M. (1996). Ecotype-specific expression of a flowering mutant phenotype in *Arabidopsis thaliana*. *Plant Physiol.* 111 641–644 12226317 10.1104/pp.111.2.641 PMC157877

[B54] Schatlowski N. Creasey K. Goodrich J. Schubert D. (2008). Keeping plants in shape: polycomb-group genes and histone methylation. *Semin. Cell Dev. Biol.* 19 547–553 10.1016/j.semcdb.2008.07.019 18718547

[B55] Schatlowski N. Stahl Y. Hohenstatt M. L. Goodrich J. Schubert D. (2010). The CURLY LEAF interacting protein BLISTER controls expression of polycomb-group target genes and cellular differentiation of *Arabidopsis thaliana*. *Plant Cell* 22 2291–2305 10.1105/tpc.109.073403 20647345 PMC2929108

[B56] Schubert D. Primavesi L. Bishopp A. Roberts G. Doonan J. Jenuwein T. (2006). Silencing by plant polycomb-group genes requires dispersed trimethylation of histone H3 at lysine 27. *EMBO J.* 25 4638–4649 10.1038/sj.emboj.7601311 16957776 PMC1590001

[B57] Schuettengruber B. Martinez A. M. Iovino N. Cavalli G. (2011). Trithorax group proteins: switching genes on and keeping them active. *Nat. Rev. Mol. Cell Biol.* 12 799–814 10.1038/nrm3230 22108599

[B58] Schwartz Y. B. Pirrotta V. (2008). Polycomb complexes and epigenetic states. *Curr. Opin. Cell Biol.* 20 266–273 10.1016/j.ceb.2008.03.002 18439810

[B59] Semiarti E. Ueno Y. Tsukaya H. Iwakawa H. Machida C. Machida Y. (2001). The ASYMMETRIC LEAVES2 gene of *Arabidopsis* *thaliana* regulates formation of a symmetric lamina, establishment of venation and repression of meristem-related homeobox genes in leaves. *Development* 128 1771–1783 11311158 10.1242/dev.128.10.1771

[B60] Sheldon C. C. Burn J. E. Perez P. P. Metzger J. Edwards J. A. Peacock W. J. (1999). The FLF MADS box gene: a repressor of flowering in *Arabidopsis* regulated by vernalization and methylation. *Plant Cell* 11 445–458 10072403 10.1105/tpc.11.3.445 PMC144185

[B61] Stone S. L. Kwong L. W. Yee K. M. Pelletier J. Lepiniec L. Fischer R. L. (2001). LEAFY COTYLEDON2 encodes a B3 domain transcription factor that induces embryo development. *Proc. Natl. Acad. Sci. U.S.A.* 98 11806–11811 10.1073/pnas.201413498 11573014 PMC58812

[B62] Sun Y. Zhou Q. Zhang W. Fu Y. Huang H. (2002). ASYMMETRIC LEAVES1, an *Arabidopsis* gene that is involved in the control of cell differentiation in leaves. *Planta* 214 694–702 10.1007/s004250100673 11882937

[B63] Suzuki M. Wang H. H. McCarty D. R. (2007). Repression of the LEAFY COTYLEDON 1/B3 regulatory network in plant embryo development by VP1/ABSCISIC ACID INSENSITIVE 3-LIKE B3 genes. *Plant Physiol.* 143 902–911 10.1104/pp.106.092320 17158584 PMC1803726

[B64] Swiezewski S. Liu F. Magusin A. Dean C. (2009). Cold-induced silencing by long antisense transcripts of an *Arabidopsis* Polycomb target. *Nature* 462 799–802 10.1038/nature08618 20010688

[B65] Turck F. Roudier F. Farrona S. Martin-Magniette M. L. Guillaume E. Buisine N. (2007). *Arabidopsis* TFL2/LHP1 specifically associates with genes marked by trimethylation of histone H3 lysine 27. *PLoS Genet. *3:e86. 10.1371/journal.pgen.0030086 PMC1885283 17542647

[B66] Vierstra R. D. (2003). The ubiquitin/26S proteasome pathway, the complex last chapter in the life of many plant proteins. *Trends Plant Sci.* 8 135–142 10.1016/S1360-1385(03)00014-1 12663224

[B67] Weinhofer I. Hehenberger E. Roszak P. Hennig L. Kohler C. (2010). H3K27me3 profiling of the endosperm implies exclusion of polycomb group protein targeting by DNA methylation. *PLoS Genet.* 6:e1001152 10.1371/journal.pgen.1001152 PMC2951372 20949070

[B68] Whitcomb S. J. Basu A. Allis C. D. Bernstein E. (2007). Polycomb Group proteins: an evolutionary perspective. *Trends Genet.* 23 494–502 10.1016/j.tig.2007.08.006 17825942

[B69] Xu L. Shen W.-H. (2008). Polycomb silencing of KNOX genes confines shoot stem cell niches in *Arabidopsis*. *Curr. Biol.* 18 1966–1971 10.1016/j.cub.2008.11.019 19097900

[B70] Xu L. Xu Y. Dong A. Sun Y. Pi L. Xu Y. (2003). Novel as1 and as2 defects in leaf adaxial-abaxial polarity reveal the requirement for ASYMMETRIC LEAVES1 and 2 and ERECTA functions in specifying leaf adaxial identity. *Development* 130 4097–4107 10.1242/dev.00622 12874130

[B71] Xu L. Zhao Z. Dong A. Soubigou-Taconnat L. Renou J. P. Steinmetz A. (2008). Di- and tri-but not monomethylation on histone H3 lysine 36 marks active transcription of genes involved in flowering time regulation and other processes in *Arabidopsis thaliana*. *Mol. Cell Biol.* 28 1348–1360 10.1128/MCB.01607-07 18070919 PMC2258740

[B72] Yang C. Bratzel F. Hohmann N. Koch M. Turck F. Calonje M. (2013). VAL- and AtBMI1-mediated H2Aub initiate the switch from embryonic to postgerminative growth in *Arabidopsis*. *Curr. Biol.* 23 1324–1329 10.1016/j.cub.2013.05.050 23810531

[B73] Yoshida N. Yanai Y. Chen L. Kato Y. Hiratsuka J. Miwa T. (2001). EMBRYONIC FLOWER2, a novel polycomb group protein homolog, mediates shoot development and flowering in *Arabidopsis*. *Plant Cell* 13 2471–2481 11701882 10.1105/tpc.010227 PMC139465

[B74] Zhang H. Bishop B. Ringenberg W. Muir W. M. Ogas J. (2012). The CHD3 remodeler PICKLE associates with genes enriched for trimethylation of histone H3 lysine 27. *Plant Physiol.* 159 418–432 10.1104/pp.112.194878 22452853 PMC3375975

[B75] Zhang X. Bernatavichute Y. V. Cokus S. Pellegrini M. Jacobsen S. E. (2009). Genome-wide analysis of mono-, di- and trimethylation of histone H3 lysine 4 in *Arabidopsis* *thaliana*. *Genome Biol.* 10 R62 10.1186/gb-2009-10-6-r62 PMC2718496 19508735

[B76] Zhang X. Clarenz O. Cokus S. Bernatavichute Y. V. Pellegrini M. Goodrich J. (2007a). Whole-genome analysis of histoneH3 lysine 27 trimethylation in *Arabidopsis*. *PLoSBiol.* 5:e129 10.1371/journal.pbio.0050129 PMC1852588 17439305

[B77] Zhang X. Germann S. Blus B. J. Khorasanizadeh S. Gaudin V. Jacobsen S. E. (2007b). The *Arabidopsis* LHP1 protein colocalizes with histone H3 Lys27 trimethylation. *Nat. Struct. Mol. Biol.* 14 869–871 10.1038/nsmb1283 17676062

[B78] Zhang H. Rider S. D. Henderson J. T. Fountain M. Chuang K. Kandachar V. (2008). The CHD3 remodeler PICKLE promotes trimethylation of histone H3 lysine 27. *J. Biol. Chem.* 283 22637–22648 10.1074/jbc.M802129200 18539592 PMC2504882

[B79] Zhao Z. Yu Y. Meyer D. Wu C. Shen W. H. (2005). Prevention of early flowering by expression of FLOWERING LOCUS C requires methylation of histone H3 K36. *Nat. Cell Biol.* 7 1256–1260 10.1038/ncb1329 16299497

